# Pediatric Heart Failure Inpatient Mortality: A Cross-Sectional Analysis

**DOI:** 10.7759/cureus.26721

**Published:** 2022-07-10

**Authors:** Ebenezer O Adebiyi, Ehizogie Edigin, Hafeez Shaka, Juanita Hunter, Sethuraman Swaminathan

**Affiliations:** 1 Pediatrics, University of Miami Miller School of Medicine, Miami, USA; 2 Rheumatology, Loma Linda University Health, Loma Linda, USA; 3 Internal Medicine, John H. Stroger, Jr. Hospital of Cook County, Chicago, USA; 4 Pediatric Cardiology, University of Miami Miller School of Medicine, Miami, USA

**Keywords:** congenital heart disease, sepsis, pediatrics, hypoplastic left heart syndrome, heart failure

## Abstract

Background

Heart failure constitutes significant morbidity and mortality among the pediatric population. Few data exist on the prevalence and mortality rate of pediatric heart failure (pHF) in the United States.

Objectives

This study aimed to determine the in-hospital mortality and the principal diagnoses in pediatric patients with heart failure who died while being hospitalized in the United States.

Methods

This is a retrospective cross-sectional study using data from the 2019 Kid Inpatient Database (KID). The KID contained data on hospitalized children below 21 years of age. Using Stata 17 software (StataCorp LLC, College Station, Texas), the data were searched for heart failure diagnoses using International Classification of Diseases 10th revision Clinical Modification (ICD-10-CM) codes. By using the “rank” command in Stata, the most common principal diagnoses were placed in descending order of frequency, and these were further divided into different ICD-10 code categories.

Results

There were 16,206 pHF admissions in 2019. Of these admissions, 1,023 (6.31%) patients died. The top five principal ICD 10 code categories among all pHF deaths in descending order were circulatory system (17.95%), congenital/chromosomal abnormalities (17.43%), respiratory system (10.28%), infectious diseases (9.24%, and perinatal diseases (7.90%). Among all pHF deaths, sepsis of unspecified organisms (5.14%), hypoplastic left heart syndrome (HLHS) (3.19%), and acute respiratory failure with hypoxia (3.14%) were the most common primary diagnoses.

Conclusion and significance

Pediatric heart failure in-hospital overall mortality is 6.31%, and sepsis of unspecified organisms, HLHS, and acute respiratory failure are the most common principal diagnoses among these children. Preventive measures and prompt treatment of infections are paramount to reducing pHF mortality.

## Introduction

Heart failure (HF) causes significant morbidity and mortality in children [1, 2]. Though there is extensive research on adult heart failure, pediatric heart failure (pHF) has received less attention in terms of etiology, management, and outcomes. While adult HF is mostly due to cardiovascular conditions such as hypertension and coronary artery disease, pHF is caused by heterogeneous factors, including cardiovascular and noncardiovascular abnormalities [3].

Congenital heart disease (CHD), which occurs in approximately eight per 1000 live births, is the most common cause of pHF [4, 5]. Children with CHD may have underlying anatomic and conduction abnormalities, ongoing volume, and pressure overload, or have myocardial reperfusion injury, which increases their risk for HF [6]. A significantly large post-tricuspid left to right shunts such as large ventricular septal defects or moderate to large patent ductus arteriosus often cause pulmonary over-circulation and result in congestive HF despite normal systolic left ventricular function. Likewise, severe left-sided obstructions such as critical aortic stenosis or severe aortic coarctation increase left heart pressure and rapidly cause heart failure if not corrected early. Though CHD remains an important cause of pHF, recent advances in surgical and catheter techniques for CHD repair, along with improvements in intensive care management, are increasing the survivorship of many children with CHD into adulthood [7]. 

Cardiomyopathy is another important cause of pHF. It accounts for 1.13 cases per 100,000 children in the United States [8]. It is a leading cause of heart transplantation in children older than one year [8]. Pediatric cardiomyopathies can result from a variety of factors such as coronary artery abnormalities, toxins, infections, or from underlying comorbid conditions. The influence of genetic mutations in the etiology of cardiomyopathies is becoming more apparent with the increasing availability and accuracy of genetic testing. Pediatric cardiomyopathies typically have phenotypic characteristics [9]. For example, dilated cardiomyopathy manifests as HF with reduced ejection fraction. Cardiomyopathy-associated HF manifests at any age, including in the fetus [8].

Pediatric HF (pHF) related in-hospital mortality was estimated to be 7.3% about ten years ago [10]. With significant improvement in both operative and perioperative care of children with CHD as well as management of pHF over the last decade, we hypothesize that in-hospital pHF mortality is decreasing in the United States. Also, there is little body of evidence on the primary diagnoses among pediatric patients with HF admitted into the hospitals in the U.S. This study used a nationally representative sample to assess the current pHF in-hospital mortality rate and identify the common principal diagnoses in children with heart failure who died during their admissions. The results of this study will provide a better understanding of the current burden of pHF in the United States and highlight areas that can be targeted to improve outcomes for children with heart failure in the United States.

## Materials and methods

Study design and sample

This was a retrospective cross-sectional study using data from the 2019 Kid Inpatient Database (KID), a Healthcare Cost and Utilization Project (HCUP) database by the Agency for Healthcare Research and Quality. The KID is the largest public database on pediatric hospital admissions in the United States, with a rough weight of seven million hospitalizations per year [11]. It includes all-payer admissions from children’s hospitals in 48 states of the U.S as well as the District of Columbia. These hospitals are either teaching, non-teaching, rural or urban. The data were abstracted from administrative data used for billing. Hospitalized children younger than 21 years of age who had a principal or secondary diagnosis of heart failure were included in this study. 

Outcomes

The primary outcome of this study was to determine the in-hospital mortality of pHF in 2019. Our secondary outcome was to determine the most common primary diagnoses in children with heart failure who died while being hospitalized.

Clinical variables

Children with heart failure were identified using the International Classification of Diseases 10th revision (ICD-10) codes. There are up to 19 diagnoses relating to heart failure included in this study (see appendix). Previously published studies identified heart failure in children using the ICD-9 version of these codes [10]. The demographic data included are age, sex, race/ethnicity, and primary payer types. The hospital characteristics included are hospital bed size, ownership, teaching status, and location. Other variables included were total hospital charges and length of hospital stay. Comorbidity burden was assessed using the Deyo adaptation of the Charlson Comorbidity Index (CCI). The CCI was grouped into three groups, with higher groups associated with an increased risk of mortality [12].

Statistical analysis

The Stata 17 software (StataCorp LLC, College Station, Texas) was used for the analyses in this study. Descriptive characteristics were first generated for the variables and were expressed as either means or frequency (percentage). Continuous and categorical variables were compared using the student t-test and the Fisher exact test respectively. In-hospital mortality was expressed as a percentage of deaths among all pHF admissions. All reported p-values were two-tailed, and values less than 0.05 were considered significant. Using the “rank” command, Stata provided the most common principal diagnoses among all ages placed in descending order of frequency. These were divided into different ICD 10 code categories. The sample was further divided into separate age groups, and the same analyses were performed to generate the most common principal diagnosis among the different age groups. Those principal diagnoses relating to the place of birth and type of delivery were excluded from the ranking as they do not belong to any pathologic condition. All analyses in this study considered the complex sampling design of the HCUP database including stratification, clustering, weighting as well as the selection of subpopulations of interest. 

## Results

There were 16,206 pHF hospitalizations from a total weight of seven million U.S pediatric hospitalizations in 2019. Of these patients, 1,023 (6.31%) died during their admission. Among these, males (550, 53.65%) and whites (430, 44.65%) had higher deaths. The mean age at death was 5.37 years (SE 0.31). Most of the patients were children less than two years (596, 58%) compared with children ages 2-12 years (17.72%) or those between 13 and 21 years of age (23.95%). The average length of stay (LOS) and total hospital charges were 33.77 days and $1,037,099, respectively. Most of the patients had Medicaid (611, 59.7%) or private insurance (366, 35.7%). The majority of the patients were admitted to large hospital bed sizes (70.26%) compared with medium (20.5%) or small (9.24%) hospital bed sizes. Compared to non-teaching hospitals (3.14%), most admissions occurred at teaching hospitals (96.86%). Most patients had one comorbidity (609, 59.5%) (Table 1).

**Table 1:** Demographic and clinical characteristics of pediatric hospital admissions for heart failure in 2019 LOS - length of stay

	Died		Alive		p-value
	n	%	n	%	
Total number	1023	6.31	15174	93.67	
Mean age, years (SE)	5.37 (0.31)		6.23 (0.19)		
Age groups (years)					0.001
< 2	596	58.33	7477	49.28	
2 -12	181	17.72	3562	23.48	
13 - 20	245	23.95	4137	27.27	
Sex					0.0143
Female	473	46.34	7787	51.32	
Male	550	53.65	7386	48.68	
Race					0.006
White	430	44.65	6817	44.93	
Black	221	22.97	3005	19.81	
Hispanic	189	19.7	3618	23.85	
Asian/Pacific Islander	31	3.29	644	4.25	
Other races	90	8.95	1084	6.36	
Insurance					0.001
Medicaid	572	54.76	8755	57.7	
Private	337	33.71	5885	38.13	
Self-pay	39	3.91	389	2.57	
Others	64	6.3	1130	7.45	
Hospital region					0.87
Northeast	136	13.35	1993	13.14	
Midwest	208	20.48	3354	22.11	
South	433	42.39	6391	42.12	
West	243	23.78	3435	22.64	
Hospital bed size					0.58
Small	94	9.24	1632	10.76	
Medium	209	20.5	3069	20.23	
Large	718	70.26	10471	69.01	
Hospital teaching status					0.839
Teaching	990	96.86	455	3	
Non-teaching	32	3.14	14718	97	
Charlson Comorbidity Index					0.001
1	608	59.5	10798	70.57	
2	206	20.22	2339	15.42	
3 and above	206	20.21	2031	13.39	
Mean LOS (days)	33.77		20.5		0.001
Mean total hospital charges ($)	1,037,099		435,270		0.001

**Figure 1:**
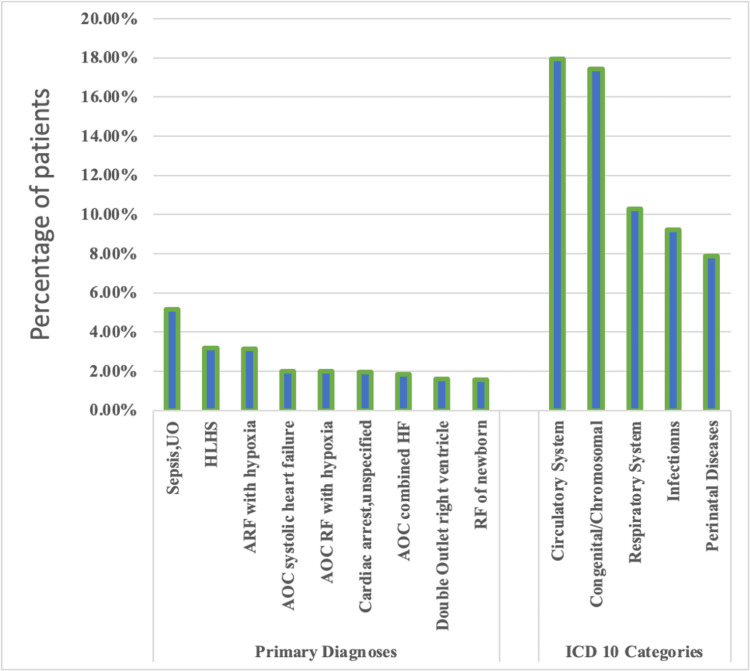
Distribution of primary diagnoses and ICD 10 code categories among pediatric heart failure deaths AOC - acute on chronic; RF - respiratory failure; HF - heart failure, ICD 10 - International Classification of Diseases 10th revision

The top five principal ICD 10 code categories among all pHF deaths in descending order were the circulatory system (17.95%), congenital/chromosomal abnormalities (17.43%), respiratory system (10.28%), infectious diseases (9.24%), and perinatal diseases (7.90%). Among all pHF deaths, sepsis of unspecified organism (5.14%), hypoplastic left heart syndrome (HLHS) (3.19%), and acute respiratory failure with hypoxia (3.14%) were the most common primary diagnoses (Figure 1).

Hypoplastic left heart syndrome (3.65%), respiratory failure of a newborn (2.70%), and double outlet right ventricle (2.51%) were among the most common primary diagnoses in children below two years of age. 

Sepsis of unspecified organism and acute on chronic systolic heart failure were the top two diagnoses among children ages above two years who died of HF. Whiles sepsis constituted 8% of all primary diagnoses among children between two to 12 years, it accounted for 11.59% among children between 13 to 20 years. Acute on chronic combined systolic and diastolic HF (4.54%) ranked third among children ages 2-12 years, whereas acute respiratory failure (3.79%) ranked third among all primary diagnoses in children ages 2-20 years with HF who died during their hospital admissions (Table 2). 

**Table 2:** Primary diagnoses among pediatric heart failure-related in-hospital deaths according to different age groups

Age (years)	Primary diagnoses	n	%
Less than 2	Hypoplastic left heart syndrome	21	3.65
	Respiratory failure of newborn	16	2.7
	Double outlet right ventricle	15	2.51
2 - 12	Sepsis, unspecified organism	14	8
	Acute on chronic systolic heart failure	9	5.19
	Acute on chronic combined systolic and diastolic HF	8	4.54
	Hypoplastic left heart syndrome	8	4.51
13 - 20	Sepsis, unspecified organism	28	11.59
	Acute on chronic systolic heart failure	10	3.99
	Acute respiratory failure	9	3.79

## Discussion

Based on our study, the overall in-hospital mortality among pHF is 6.3%. Sepsis, HLHS, and acute respiratory failure are the most common principal diagnoses among these patients. 

The overall mortality rate in our study was slightly lower when compared to the study by Rossano et al., who noted a mortality rate of 7.3% among pHF admissions [10]. That study obtained information from 1999 to 2006 from the same database we have used for our current study. Several factors may contribute to the decrease in mortality of patients with pHF over the last decade. CHD is the most prevalent comorbid condition in pHF, and the outcome of CHD continues to improve due to the early surgical and catheter interventions. An Australian study among 1196 children by Massin et al. found that only 10 percent of children with both congenital and acquired heart disease developed HF [13]. The authors of that study concluded that the low incidence of heart failure is primarily due to the early introduction of surgical and catheter interventions [13]. Also, there is increasing use of medical and surgical treatment modalities, which have proven successful in adult heart failure management among children with HF [14]. Various circulatory support devices, such as extracorporeal membrane oxygenation (ECMO), as well as ventricular support devices, now play significant roles in the management of advanced HF in children. These devices are also being used as a bridge to heart transplantation in some centers [14]. Also, newer medications such as ivabradine and sacubitril/valsartan that have proven effective in the adult population are being introduced in pHF management. Many other similar candidate drugs are undergoing clinical trials [15].

To our knowledge, this is the first study to evaluate the primary diagnoses among children with heart failure who died during hospital admissions in the U.S. We have shown that sepsis of unspecified organism, HLHS, and acute respiratory failure with hypoxia in descending order, constituted the most common principal diagnoses among children with heart failure who died while being hospitalized. Children with HF may have varying degrees of immunodeficiency status [16], and therefore sepsis is a significant risk factor for mortality among these children [17]. The increase in the mortality rate of patients with combined HF and sepsis is as high as 90% [18]. HLHS has the highest mortality among all congenital heart defects during the first year of life [19]. The complex physiology in HLHS often causes systemic and coronary hypoperfusion resulting in tissue hypoxia, metabolic acidosis, and death. Using the national inpatient sample and KID, Hamzah et al. showed that in-hospital mortality among neonates with HLHS decreased from 25.3% two decades ago compared to 20.6% in the last eight years [19]. Though survival for HLHS has improved in recent years, the one-year survival rate remains between 20 and 60% [20]. Similarly, respiratory failure is a well-known major cause of morbidity and mortality among hospitalized infants and young children in many developed countries [21].

The large population included in the KID data minimizes the likelihood of beta error. Also, KID provides a larger sample size based on nationally representative samples of hospitalized patients. Hence, our results should broadly reflect pediatric patients with HF admitted to hospitals in the U.S. 

This study is limited due to the nature of the data and the method with which they were collected. The HCUP KID is an administrative database where diagnoses were coded using the ICD codes. There is a possibility of misclassification in the process of selecting diagnoses due to human errors. Also, clinical data such as vital signs, medications, and echocardiology reports, which are important to properly characterize the patients, are lacking in the database used in this study.

## Conclusions

In conclusion, there is decreasing pHF in-patient overall mortality in the United States. Several factors, such as improved care for CHD as well as advances in the management of pediatric heart failure, may be contributing to this downward trend of pHF in-hospital deaths. However, sepsis and HLHS are still significant causes of morbidity and mortality in pHF. Preventive measures and prompt treatment of infections are paramount to reducing pHF in-hospital mortality. Also, strategies that improve HLHS health outcomes, such as single ventricle monitoring programs, will significantly reduce overall mortality in pHF.

## Appendices

**Table 3:** ICD 10 diagnosis codes used in the analysis ICD 10 - International Classification of Diseases 10th revision

Heart failure diagnoses	
I50.1	Left ventricular failure
I50.20	Unspecified systolic (congestive) heart failure
I50.21	Acute systolic (congestive) heart failure
I50.22	Chronic systolic (congestive) heart failure
I50.23	Acute on chronic systolic (congestive) heart failure
I50.30	Unspecified diastolic(congestive)heart failure
I50.31	Acute diastolic (congestive) heart failure
I50.32	Chronic diastolic (congestive) heart failure
I50.33	Acute on chronic diastolic (congestive) heart failure
I50.40	Unspecified combined systolic(congestive) and diastolic (congestive) heart failure
I50.41	Acute combined (congestive) and diastolic (congestive) heart failure
I50.42	Chronic combined systolic (congestive) and diastolic (congestive) heart failure
I50.43	Acute on chronic combined systolic (congestive) and diastolic (congestive) heart failure
I50.9	Heart failure, unspecified
I09.81	Rheumatic heart failure
I11.0	Hypertensive heart disease with heart failure
I13.0	Hypertensive heart and chronic kidney disease (stage 1-4) with heart failure
I13.2	Hypertensive heart and chronic kidney disease (stage 5) with heart failure
P29.0	Neonatal cardiac failure
Primary diagnoses	
Q23.4	Hypoplastic left heart syndrome
P28.5	Respiratory failure of newborn
Q20.1	Double outlet right ventricle
A41.9	Sepsis, unspecified organism
J96.01	Acute respiratory failure with hypoxia
J96.21	Acute on chronic respiratory failure with hypoxia
I46.9	Cardiac arrest, cause unspecified
